# Determinants of community structure of zooplankton in heavily polluted river ecosystems

**DOI:** 10.1038/srep22043

**Published:** 2016-02-25

**Authors:** Wei Xiong, Jie Li, Yiyong Chen, Baoqing Shan, Weimin Wang, Aibin Zhan

**Affiliations:** 1 Research Center for Eco-Environmental Sciences, Chinese Academy of Sciences, 18 Shuangqing Road, Haidian District, Beijing 100085, China; 2 College of Fisheries, Huazhong Agricultural University, 1 Shizishan Street, Wuhan 430070, China

## Abstract

River ecosystems are among the most affected habitats globally by human activities, such as the release of chemical pollutants. However, it remains largely unknown how and to what extent many communities such as zooplankton are affected by these environmental stressors in river ecosystems. Here, we aim to determine major factors responsible for shaping community structure of zooplankton in heavily polluted river ecosystems. Specially, we use rotifers in the Haihe River Basin (HRB) in North China as a case study to test the hypothesis that species sorting (i.e. species are “filtered” by environmental factors and occur at environmental suitable sites) plays a key role in determining community structure at the basin level. Based on an analysis of 94 sites across the plain region of HRB, we found evidence that both local and regional factors could affect rotifer community structure. Interestingly, further analyses indicated that local factors played a more important role in determining community structure. Thus, our results support the species sorting hypothesis in highly polluted rivers, suggesting that local environmental constraints, such as environmental pollution caused by human activities, can be stronger than dispersal limitation caused by regional factors to shape local community structure of zooplankton at the basin level.

Frequent disturbance derived from increasing anthropogenic activities has made freshwater ecosystems among the most threatened habitats globally^1^. Among several types of freshwater ecosystems, rivers are among the most polluted by chemicals^2^. As rivers usually occupy the lowest-lying areas on the landscape, they sink various chemical pollutions from both point and non-point sources^3^. Chemical pollution has become a serious problem in river ecosystems globally, but is particularly acute in developing countries such as China^2^. Almost one-third of streams/rivers in China have been recorded as polluted or highly polluted (Report on the State of the Environment in China, 2013).

As rivers are important aquatic ecosystems supporting diverse life forms, a high level of pressure from interacting stressors including chemical pollution has driven extinction rates of freshwater organisms much higher than those for terrestrial species^4^. By 2012, more than 4600 freshwater animals were identified as threatened or recently extinct, accounting for more than 25% of all identified freshwater animals^5^. It is well-known that chemical pollution can directly and/or indirectly lead to biodiversity loss and/or re-distribution in aquatic ecosystems^6^,^7^,^8^. However, it remains largely unknown how and to what degree environmental changes derived from chemical pollution influence community structure and geographical distribution of biodiversity, particularly zooplankton, in river ecosystems.

The mechanisms underlying variability in species composition and geographical distributions of zooplankton biodiversity are complex in running water ecosystems^9^,^10^,^11^,^12^. Some studies showed that the dispersal capacity of organisms determined local community structure^13^,^14^, while others illustrated that local environmental factors, such as water temperature, pH, salinity, trophic state or combinations of these factors were responsible for shaping local community structure (i.e. species sorting hypothesis)^15^,^16^. A comprehensive review of available evidence suggested that many factors, including spatial scale and extent, dispersal rates, and environmental gradient lengths may explain the inconsistent results in different studies^17^. Based on the conceptual framework constructed by Heino *et al.*^18^, environmental factors are likely to be most important for shaping community structure (i.e. species sorting hypothesis) when dispersal rates are intermediate (i.e. at a basin level)^19^. However, the species sorting hypothesis remains largely untested in zooplankton communities in river ecosystems affected by chemical pollution. Here we use rotifer communities in the most polluted river in China, the Haihe River Basin, to test the species sorting hypothesis at the basin scale.

Rotifera is one of the dominant microscopic animal assemblages widely distributed in all types of freshwater and brackish water bodies^20^. They play an important role in aquatic food webs by producing and structuring the matter, energy and information fluxes in aquatic ecosystems^21^. Empirical studies have shown that rotifer communities were sensitive to environmental changes, such as nutrient importing and pH and water temperature changes^16^,^22^,^23^. Environmental change-induced fluctuation of rotifer community could result in an alternation of food webs directly, which may further lead to significant influence on aquatic ecosystem stability. Consequently, rotifer communities are usually considered as effective indicators for assessing the health status of aquatic ecosystems^23^,^24^. Collectively, rotifers represent a good model not only to test the hypothesis motioned above, but also to understand how environmental changes, such as chemical pollution, influence aquatic ecosystem stability and functioning.

The Haihe River Basin (HRB) is one of the largest water systems in the North China. The basin consists of more than 300 tributaries that spread out like a palm-leaf over a large area before converging near Tianjin (Fig. 1). It covers 318,000 km^2^, including the fastest growing economic regions, such as Beijing, Tianjing, and more than 120, 000 km^2^ of farmland (Bulletin of water resources in HRB, 1998). Chemical pollutants, particularly nitrogen derived from both point (e.g. waste water releases from a large number of industries and cities) and non-point sources (e.g. those derived from farmlands where chemical fertilizers and pesticides are commonly used), have largely released into HRB^25^. Consequently, most rivers in HRB have become highly eutrophic over the past two decades^26^,^27^ (also see data in Supplementary Table S2).

Here we investigated the community structure of planktonic rotifers in the plain region of HRB, where water pollution represents a huge threat to biodiversity and ecosystem functioning. Using planktonic rotifers as a model, we integrated local biotic and abiotic factors and spatial configuration to study the key factors responsible for local community structure in heavily polluted river ecosystems. Further, we tested the hypothesis that species sorting plays a key role in shaping zooplankton community structure in highly polluted river ecosystems at the basin level.

## Results

### Community structure

A total of 91 rotifer species was detected across all 94 sampling sites. Species richness slightly decreased from zone I to zone III, with 65, 57 and 50 species in zone I–III, respectively (Fig. 2). Only 29 species (31.7%) were common among the three zones, while 36 species (39.6%) were zone-specific (Fig. 2). As expected, the largest and smallest number of zone-specific species was detected in zone I (i.e. upper stream; 21 species, 23.1%) and zone III (lower reaches of rivers; 7 species, 7.7%), respectively. In general, a greater number of species were shared between neighboring sites than between geographically isolated sites. For example, the number of common species between zones I and II was 9, larger than that (6) between zones I and III (Fig. 2). When the four major diversity indices, including species richness, total abundance, Pielou’s evenness and Shannon-Wiener diversity index, were subjected to the non-parametric Mann-Whitney *U* test, we did not find statistical difference between zones (*P* > 0.05 for all pairs). In addition, the statistical percentiles also showed a coincident result, as there appeared similar median values and 25^th^ and 75^th^ percentiles (Fig. 3).

However, we found a high level of dissimilarity of community structure among sampling sites at both intra- (Supplementary Table S3) and inter-zone levels (Table 1). The average Bray-Curtis similarity of rotifer communities in each zone was low (13.44, 19.22 and 24.49 in zones I–III, respectively). In general, the intra-zone dissimilarity of rotifer communities decreased from zone I to III (Supplementary Table S3). When the abundance of each species was compared between zones, we detected a significant difference between zones I and II (ANOSIM, *P* < 0.05; Table 1). Although we did not find significant difference between zones II and III, the average dissimilarity between these two zones was as high as 78.5% (SIMPER, Table 1). Compared to relatively rare species, the change of dominant species abundance was responsible for approximately half of the dissimilarity. For example, the top seven dominant species contributed to 47.4% and 55.5% of the average dissimilarity between zones I and II, and between zones II and III, respectively (Table 1). However, further analysis based on SIMPER showed that the contribution of single taxa to spatial dissimilarity was relative low (SIMPER values < 11.3%; Table 1), suggesting that the change of abundance of a group species, rather than the presence/absence of particular species, was responsible for the detected high level of dissimilarity between zones.

### Factors responsible for the observed community structure

Regression analyses indicated that local abiotic factors (i.e. environmental factors), rather than biotic (i.e. abundance of protozoa and crustacea) and regional effects (i.e. attitude, longitude, altitude and geographical distance), were largely responsible for the observed difference of the total abundance and species richness among sampling sites (Table 2). Briefly, total nitrogen was negatively related to the total abundance and species richness, while water temperature was positively related to the total abundance.

The ordination of redundancy analysis (RDA) showed that all 13 factors were able to explain 17.0% of the total variability of community composition across all sampling sites (sum of all canonical variables = 0.125; *F* = 2.076, *P* = 0.002 for all canonical axes), and the five local factors, including total nitrogen (TN), ammonia nitrogen (NH_4_-N), total phosphorus (TP), soluble reactive phosphorus (SRP) and water temperature (T), as well as one regional factor (latitude, one factor responsible for dispersal limitation, see the Discussion section), were the leading factors responsible for shaping patterns of rotifer communities based on the Monte Carlo permutation tests of the significance of all factors (Lambda-1; Table 3, Fig. 4). After the addition of total nitrogen to the ordination, only latitude and water temperature explained any significance amount of remaining variation (*P* < 0.05; Table 3). In addition, total nitrogen was negatively correlated to abundance of most rotifer species (Fig. 4A). Taking dominant species for example (Fig. 4B,C), when we ordered sampling sites based on total nitrogen, *Polyarthra trigla* abundance decreased with increasing total nitrogen. All these results suggest that both local factors (i.e. species sorting) and regional factors (i.e. dispersal limitation) were responsible for the observed patterns of rotifer communities. We therefore further explored the relative role of these two categories of factors.

### Relative roles of local versus regional effects

The relative role of these two categories of factors was explored using variance partitioning procedure based on partial redundancy analysis (pRDA). For the 17.0% variability explained by all 13 variables, our pRDA analyses showed that nine local factors explained 10.0% (58.8% of the variability explained in the model) of the total variability in rotifer composition, and this part of variability could not be explained by regional factors (Fig. 5). Four regional factors totally explained 4.5% (26.5% of the variability explained in the model) of variability. Similarly, this part of variability could not be explained by local factors.

In summary, though local and regional factors were included in the significant explanatory of detected distribution patterns, the former (i.e. species sorting) played a more important role in driving geographical distribution of rotifer biodiversity based on multiple analyses.

## Discussion

Multiple studies illustrated that the relative roles of local and regional factors could largely differ in governing geographical distribution of community biodiversity. For example, the community structure of microorganisms and phytoplankton was more environmentally controlled, while regional effects had significant influence on fish and crustacean communities^28^. Among several factors responsible for the mixed results derived from different studies, two common ones were identified: geographical scale and dispersal capacity of species^11^,^14^,^19^,^29^,^30^. In general, local environmental effects were strong at the basin level but weak in relatively smaller scale such as the river/stream level^19^. At the same scale, in general the community structure of passive dispersers such as diatoms and bryophytes was influenced by local environmental factors when compared to actively dispersing invertebrates^14^. In addition, for passive dispersers, small-bodied groups showed weaker influence derived from local environments than large-bodied groups, suggesting a decreasing impact of dispersal limitation with decreasing body size^11^,^30^. Several biological characteristics of rotifers, such as microscopic body size, dormant stage and possible parthenogenesis reproductive model (for example Monogonont rotifers, to which most species belong in this study, Supplementary Table S1) make them as easily colonized organisms across a wide range of habitats^31^,^32^. The dispersal of planktonic rotifers in HRB occurs primarily along river flows. Long-distance migration by wind, birds and/or human movement may also causally happen, but such a type of dispersal was considered as a weaker force when compared to a high level of dispersal advected by flows^33^. Given the different environmental factors (Supplementary Table S1) and limited regional dispersal at the basin level in HRB, local environmental factors could be strong enough to shape community structure of rotifers. Our multiple analyses support this expectation and approved the species sorting hypothesis in highly polluted river ecosystems. However, further investigation is needed to elucidate consequences of the two competing forces (i.e. species sorting versus dispersal) at smaller geographical scales such as the stream/river level.

**Figure 1:**
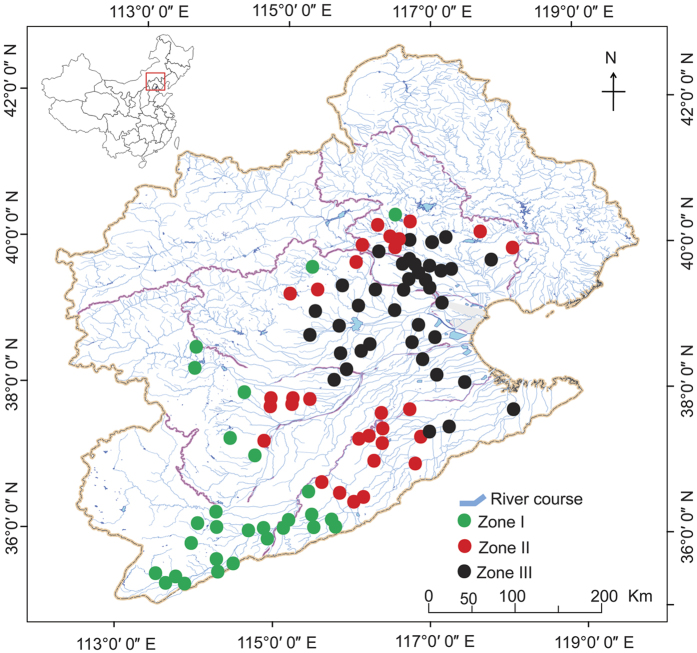
Sampling locations across the plain region of the Haihe River Basin. The plain region was divided into three zones, i.e. zones I–III that are located at upper, middle and lower reaches of rivers, respectively. Also, the three zones correspond to different altitudes: 40–369, 21–39, and 6–20 meters above sea level for zones I–III, respectively. All figures were made by ArcGIS version 10.0 (ESRI Company, USA).

**Figure 2:**
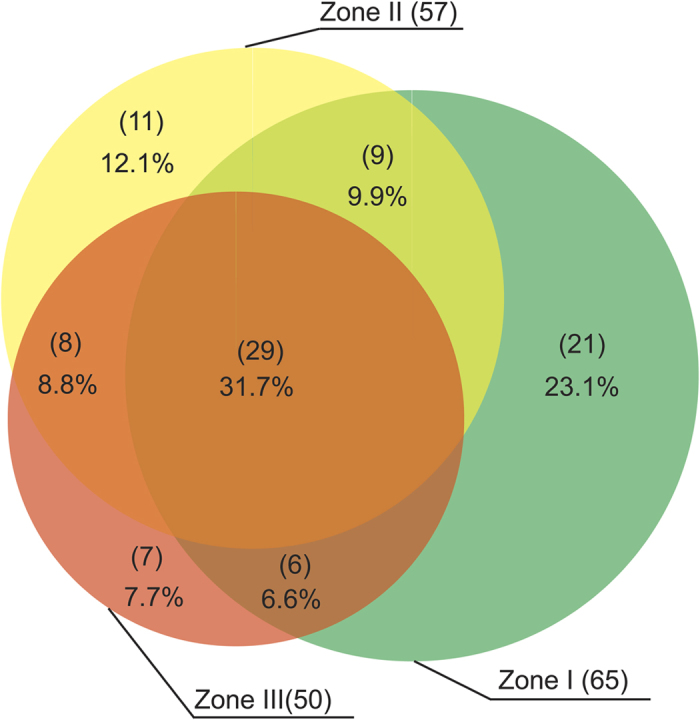
The geographical distribution (Venn diagram) of rotifer species in the plain region of the Haihe River Basin. The numbers in brackets indicate the number of rotifer species detected, while the percentage depicts the proportion of species in the pool of the recorded 91 species from all three zones.

**Figure 3:**
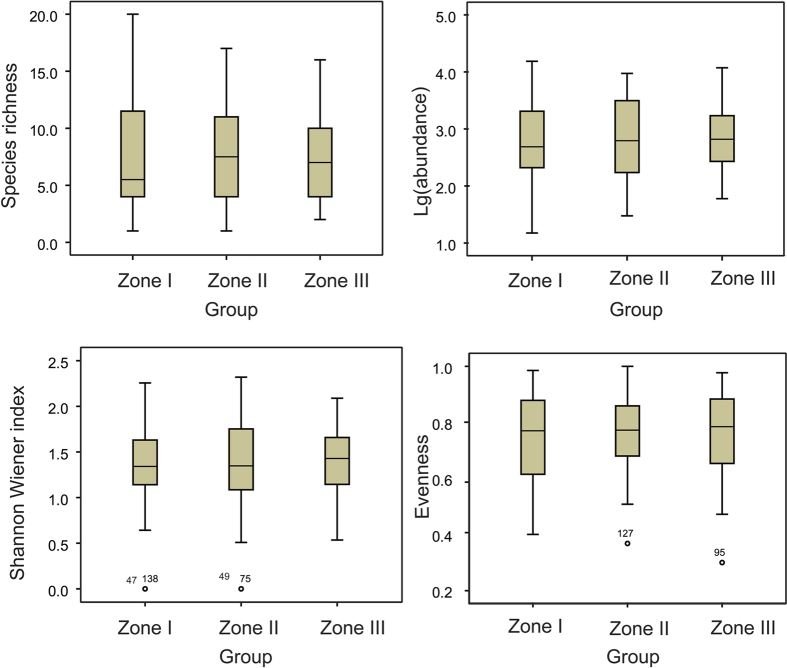
Boxplots of the diversity indices of rotifer communities in the three zones. Boxes, central bars and solid lines represent the interquartile range, the median and the data range, respectively. The outilers are circles lying outside 1.5 times the interquartile range.

**Figure 4:**
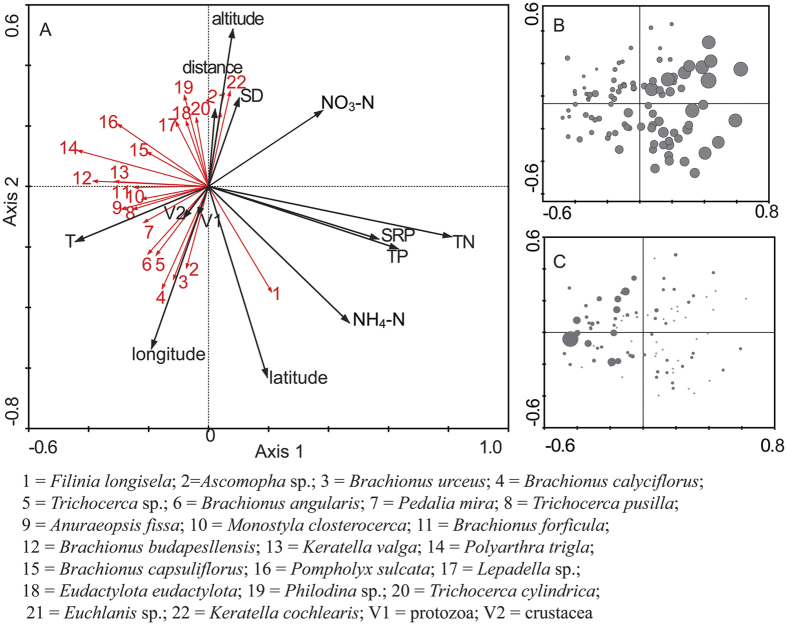
Ordination biplots based on the redundancy analysis (RDA) of rotifer communities (**A**), ordination of sampling sites based on the total nitrogen (**B**) and the corresponding *polyarthra trigla* abundance in sampling sites (**C**) in the plain region of the Haihe River Basin. Scores of rotifer species and environmental variables in Figure A were scaled to fit the sample ordination. Species weakly associated with the first two axes (with fitness < 5%) were omitted from the ordination for clarity. Arrows in red represent rotifer taxa, while arrows in black represent measured biotic and abiotc variables. The size of circles in Figures B,C represents the relative total nitrogen concentration and the relative abundance, respectively.

**Figure 5:**
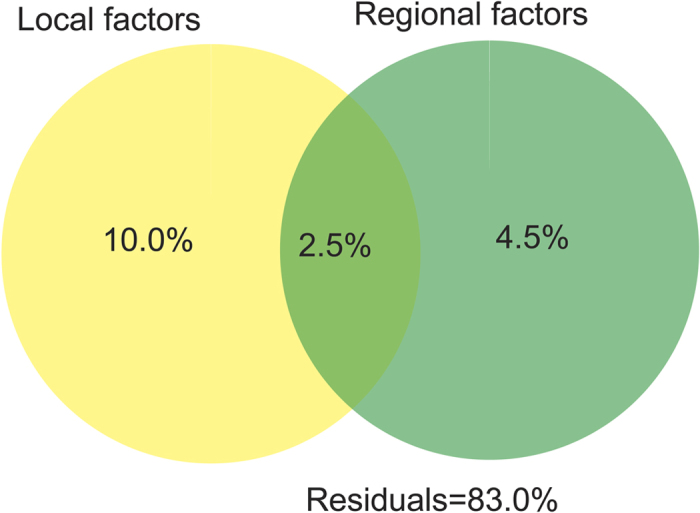
Results of variation partitioning analysis for assessing the relative importance of local and regional factors in constraining rotifer communities in the plain region of the Haihe River Basin.

Although species sorting was a stronger force in shaping local rotifer community structure in HRB, we also tested spatial structuring (regional processes) as a non-ignorable driver (Tables 2 and 3; Fig. 4). Among several regional factors, altitude was the major one (Tables 2 and 3). The dendritic nature of stream network structure and the west to east directionality of streams of HRB make streams analogous to parallel to each other and distributed along altitude (Fig. 1). A low level of overland dispersal prevails, and dispersal in watercourses is impossible in parallel headwaters. Consequently, altitude is indeed associated with dispersal limitation, and further plays a role in affecting community structure. Recent theoretical studies suggest that diversity in rivers is affected by dispersal along the dendritic landscape structure, and dispersal-driven regional processes play different roles for structuring assemblages in peripheral headwaters and in central parts of the networks^34^. In addition, water flow/discharge can be a crucial driver, which may affect passive dispersal of rotifers, especially when flow rate varies significantly in different regions. Although we did not include water flow rates here, our recent fine-scale study (i.e. single river level) suggests that dispersal advected by water flow has little effect on community structure in heavily polluted rivers (not published data).

Among all abiotic and biotic factors measured in this study, total nitrogen, which is the major chemical pollutant in HRB, was the most important factor in affecting rotifer community structure: the total abundance and species richness decreased when total nitrogen increased (Table 2). Furthermore, total nitrogen explained the largest variation of rotifer community when compared with other local factors (Table 3, Fig. 4). Studies have successfully identified the determinant role of total nitrogen on rotifer community structure^22^,^35^. However, it remains largely inconsistent in many studies on the relationships between species abundance and concentration of total nitrogen (i.e. negative or positive) and the degree to which rotifer communities influenced by total nitrogen. For example, Vakkilainen *et al.*^35^ confirmed that rotifers respond negatively to nutrient enrichment by mesocosm experiments in hypereutrophic conditions. A strong negative correlation between rotifer species number and Trophic Level Index (TLIc) was explicitly described by field work in eutrophicated lakes^36^. However, several studies indicated that nitrogen could increase rotifer density (e.g. Wang *et al.*^37^). When comparing these studies, we found different range and/or length of gradient of nitrogen concentration and corresponding trophic state in surveyed regions. For example, the total nitrogen concentration ranged from 0.48 to 8.67 mg L^−1^ in Wang *et al.*’s study, while we detected 0.6–30.0 mg/L in HRB in this study, which represents higher nutrient loading and a much longer environmental gradient length in HRB (Supplementary Table S1). Nitrogen is an essential component for the growth and development of organisms such as microalgae. The increase of nitrogen concentration at a given range may increase food availability for rotifers, leading to higher species abundances. However, excess nitrogen would turn into toxic ammonia and sharply decrease the survival rate of rotifers^38^.

Although we had evidence that species sorting played a key role in shaping rotifer community structure, our study was still limited to one season, i.e. we did not take any seasonal dynamics into account. There were two reasons to perform our analyses in only one season snapshot. Firstly, the aim of our study is to test the species sorting hypothesis at the basin level. Consequently, we only considered the effects of different biotic and abiotic factors on geographical distribution of biodiversity (i.e. spatial variation, rather than temporal/seasonal variation), akin to a large number of studies (e.g. Cottenie *et al.*^39^; Viana *et al.*^40^). Secondly, we finished sampling within 21 days for all 94 sampling sites. Our surveys on temporal variation of rotifer communities showed no significant changes in 16 representative locations within one month in the same season (unpublished data). Consequently, the effect of temporal/seasonal variation on our results in this study should be minimal. However, it remains essential to include seasonal dynamics to further test what degree the species sorting can determine the community structure of zooplankton across seasons.

Similar to many studies (e.g. Parkes & Duggan^41^), the percentage of community variation explained by measured variables was only 17.0%. However, the explanatory effect of each variable was not affected: for example, the explained variation of total nitrogen accounted for 29.4% (0.05/0.17) of the species-environment relationship explained in the model. Collectively, three factors could be responsible for the low proportion of explained variability: large geographical area, the frequent occurrence of rare species, and more importantly unmeasured environmental factors^42^,^43^. As a classical and common problem in such studies, it is impossible to measure all biotic and abiotic variables, especially in polluted rivers where many pollutants are unknown. It is possible that some undetected local variables affected rotifer community structure in HBR. Consequently, this limitation made our interpretation on local factor more conservative, especially when assessed the relative importance of species sorting. In addition, the local environmental factors that we considered in our study are known to be important structuring variables in freshwater habitats (e.g. Duggan *et al.*^16^; Wang *et al.*^37^; Faithfull *et al.*^44^), and our study confirmed these factors to be important in a highly polluted Chinese river.

In conclusion, our study clearly showed that the community structure of planktonic rotifers varied greatly in the plain region of HRB. Community structure differed not only at the inter-zone level but also at the intra-zone level, even among connected neighboring sites. Multiple analyses identified that both local and regional factors play roles in shaping local community structure of rotifers in the HRB. Although it is well documented that regional factors largely shape local community structure of zooplankton in little disturbed running waters^14^,^18^,^19^, interestingly when compared to regional factors, our study here clearly showed that local environmental factors, especially total nitrogen, played a more important role in heavily polluted river. Our results support the species sorting hypothesis at the basin level, showing that environmental pollution can be strong enough to determine local community structure of zooplankton. In addition, we found that species richness and total abundance of rotifers didn’t vary significantly between zones, while there was a high level of community dissimilarity among sampling sites, including those highly connected ones within each zone. Local abiotic factors, rather than biotic and regional effects, were largely responsible for the observed patterns. The total nitrogen was negatively related to the total abundance and species richness, while water temperature was positively related to the total abundance. Our findings highlight the necessity to consider the effects of pollution directly and/or indirectly derived from human activities on biodiversity and its geographical distributions when addressing many fundamental and conservation management issues. In addition, as biodiversity in river ecosystems is valuable, its future conservation and management will require integration of both biotic and abiotic determinant factors to establish plans and actions to halt biodiversity loss.

## Methods

### Site selection

Compared to the mountain region of HRB, severe chemical pollution derived from numerous industries, cities and farmlands has largely affected the water quality in the plain region. We therefore chose the plain region to determine the key factors that influence community structure and geographical biodiversity distribution of zooplankton. In order to perform a comprehensive sampling at a large geographical scale, the plain region was characterized based on the geographical and hydrological features of all tributaries using ArcGIS version 10.0 (ESRI Company, USA). In summary, the plain region was divided into three zones, i.e. zones I–III that are located at upper, middle and lower reaches of each river, respectively (Fig. 1). In general, chemical pollution becomes more serious from zone I to zone III, despite that several exceptions exist at some sampling sites (Supplementary Table S2). The three zones also correspond to different altitudes: 40–369, 21–39, and 6–20 meters above sea level for zones I–III, respectively. According to the gradient of chemical pollution across the plain region of HRB, a total of 94 representative sampling sites were selected out of 421 analyzed locations, including 28, 28 and 38 sites in zones I–III, respectively (Fig. 1). The 94 sampling sites covered the entire plain region (approximately 1.1 × 10^5^ km^2^).

### Sample collection and data collection

As seasonal variation of community structure is not our research aim in this study, we collected rotifer samples in one season (May–June). Rotifers in each sampling site were quantitatively collected. In summary, we collected 10 L water samples from the bottom to water surface for three times, mixed three samples and filtered through a 20 μm mesh net, and subsequently preserved in 5% formaldehyde (final concentration) with a final volume of 100 mL.

We conducted species identification using three subsamples from each sampling site. For each subsample, we took 2 mL out of the 100 mL preserved samples. Bottles were well shaken before taking subsamples to avoid sampling bias. All individuals of rotifers in each subsample were identified and counted under a microscope with formaldehyde. Rotifers were identified to the species level based on available taxonomic keys^45^. For several genera such as *Synchacta* and *Trichocerca* where taxonomic keys are not available and/or hard to identify, we identified them to the genus level. In addition, in order to investigate possible biotic interaction effects on rotifer communities, two representative groups on rotifers’ food webs, i.e. protozoa and crustacea, were counted in three subsamples collected at each sampling site. The adequacy of sampling depth was assessed using species-accumulation curves, suggesting that the species richness estimated by our methods also reached or almost reached to asymptote (Supplementary Figure S1).

Two parallel 500 mL water samples were collected simultaneously during the field sampling. One water sample was filtered through a 0.45 μm glass microfiber filter for the measurement of soluble reactive phosphorus (SRP), nitrate nitrogen (NO_3_-N), and ammonia nitrogen (NH_4_-N). Another water sample was used for total nitrogen (TN) and total phosphorus (TP) analysis. TN, NH_4_-N and NO_3_-N were determined using the alkaline potassium persulfate digestion UV spectrophotometric method, Nessler’s reagent spectrophotometry, and ultraviolet spectrophotometry, respectively. TP and SRP were measured based on the ammonium molybdate spectrophotometric method. Water temperature (T), Secchi disk depth (SD), longitude, latitude, and altitude of each site were recorded in the field. The average geographical distance between a given sampling site and the others was calculated by taking the mean based on a matrix of distance between sites. The distance was examined by function earth.dist in R library fossil^46^.

### Data analysis

Biodiversity and geographical patterns of planktonic rotifer assemblages were analyzed using non-parametric multivariate methods implemented in PRIMER version 6.0^47^. The commonly used diversity parameters, including species richness, total abundance of all rotifer species, Pielou’s evenness and Shannon-Wiener diversity index, were calculated by a statistical DIVERSE routine in PRIMER for each sampling site. The difference of diversity indices between zones was assessed using the non-parametric Mann-Whitney *U* test. Moreover, we identified dominant species from each zone according to the Dufrêne’s method: *Y* = *N*_*i*_ × *f*/*N*, where *Y* is a dominance value, *N* is the total number of individuals in a zone, *i* is the case of species *i*, and *f* is the frequency of species *i* by *f*, which is the relative frequency of occurrence of species *i* in the sites of each zone. Usually, a species with the dominance value *Y* greater than or equal to 0.02 is considered as a dominant species^48^. In addition, the abundance of all rotifer species between zones was compared using the analysis of similarity (ANOSIM). ANOSIM is a non-parametric analogue for analyzing variance and testing multivariate differences between groups^49^ based on Bray-Curtis distance and rank dissimilarity. The major species driving geographic patterns of rotifer assemblages were identified using similarity percentage analysis (SIMPER). SIMPER calculates the average dissimilarity among all pairs of samples between zones and then assesses the relative dissimilarity contributed by each species. To further assess the distribution pattern of rotifer assemblages at the intra-specific level, the similarity of rotifer assemblages among sampling sites within each zone was compared by SIMPER based on Bray-Curtis distance.

Multiple-stressor effects on biodiversity of rotifer assemblages were assessed by stepwise multiple linear regression, and data derive from each site was assessed as a separate entity. Principal component analysis (PCA) was used to identify the primary gradient of environmental variables, and first five factors were selected to involve in linear regression analysis (Supplementary Table S1, based on the eigenvalue greater than 1). Separate regression models were developed for total abundance of all species and species richness with the five factors to assess the relationship of rotifer communities and environmental variables. For PCA, the diversity metrics were log_10_-transformed, and environmental variables were standardized to zero mean and unit variance to remove the influence of differing scales of measurement. The possible relationship between community structure and local/regional factors was examined by redundancy analysis (RDA). This method was chosen because preliminary detrened correspondence analysis (DCA) on square root transformed species revealed that gradient length ranged from 2.96 to 4.99 (an estimate of β-diversity), suggesting the majority of species exhibited linear responses to the environmental variation that abundances. Forward selection and Monte Carlo permutation test (1000 permutations) were performed to determine which variables were statistically significant in determining rotifer community structure.

Partial redundancy analysis (pRDA) was used to assess the relative roles of species sorting (i.e. local effect) and geographical barriers for dispersal (i.e. regional effect) in affecting community structure. All measured 13 variables were divided into two groups-local environmental factors including TN, NH_4_-N, NO_3_-N, TP, SRP, T, SD, the total abundance of protozoa and crustacea, and regional factors including attitude, longitude, altitude and geographical distance between sampling sites. For data analysis, the species composition data was square root transformed, while the environmental data was log_10_(X + 1) transformed to improve normality and subsequently was standardized to zero mean and unit variance to remove the differing scales of measurement. Both RDA and pRDA were performed using the CANOCO 4.5 package^50^.

## Additional Information

**How to cite this article**: Xiong, W. *et al.* Determinants of community structure of zooplankton in heavily polluted river ecosystems. *Sci. Rep.* **6**, 22043; doi: 10.1038/srep22043 (2016).

## Supplementary Material

Supplementary Information

Supplementary Table S2

## Figures and Tables

**Table 1:** Results of the similarity percentage (SIMPER) analysis among the three zones and the analysis of similarity (ANOSIM) of variance between the three zones in the plain region of the Haihe River Basin.

Zone I versus Zone II (Global test, *R* = 0.038, *P* value = 0.047) Average dissimilarity = 85.30			
Species	Average abundance		Contribution%
	I	II	
***Polyarthra trigla***	463.39	445.18	10.79
***Brachionus angularis***	92.68	192.32	7.73
***Filinia maio***	157.5	71.79	6.85
***Brachionus urceus***	330	73.93	6.74
***Brachionus calyciflorus***	83.57	260.89	6.41
***Trichocerca sp.***	202.5	71.25	4.7
***Filinia longisela***	8.04	221.79	4.17
*Pompholyx sulcata*	26.25	52.5	3.49
*Philodina megalotrocha*	35.36	20.89	3.16
*Anuraeopsis fissa*	35.89	69.64	2.74
*Keratella valga*	22.5	26.25	2.63
*Trichocerca pusilla*	0	56.79	2.36
**Zone I versus Zone III (Global test, *R* = 0.130, *P* value = 0.001) Average dissimilarity = 82.89**			
**Species**	**Average abundance**		**Contribution%**
	**I**	**III**	
***Polyarthra trigla***	463.39	428.29	10.89
***Brachionus urceus***	330	131.05	8.45
***Brachionus angularis***	92.68	195.79	8.16
***Trichocerca sp.***	202.5	137.76	7.28
***Filinia maio***	157.5	112.5	6.96
***Brachionus calyciflorus***	83.57	123.55	5.78
*Anuraeopsis fissa*	35.89	112.89	3.23
*Brachionus budapesllensis*	26.25	24.87	2.99
*Keratella valga*	22.5	41.05	2.94
*Pompholyx sulcata*	26.25	9.47	2.91
*Philodina megalotrocha*	35.36	22.11	2.81
**Zone II versus Zone III (Global test, *R* = 0.053, *P* value = 0.054) Average dissimilarity: 78.52**			
**Species**	**Average abundance**		**Contribution%**
	**II**	**III**	
***Polyarthra trigla***	445.18	428.29	11.13
***Brachionus angularis***	192.32	195.79	8.68
***Brachionus urceus***	73.93	131.05	8.51
***Brachionus calyciflorus***	260.89	123.55	8.31
***Trichocerca sp.***	71.25	137.76	7.08
***Filinia maio***	71.79	112.50	6.61
***Filinia longisela***	221.79	11.84	4.8
*Keratella valga*	26.25	41.05	3.54
*Trichocerca pusilla*	56.79	24.87	3.22

Species names in bold indicate dominant species.

**Table 2:** Regression models for the total abundance of all species and species richness listed by the order of the factors involved by stepwise linear regression analysis.

	Models	*R*	*P*
Abundance	Y = 1775.4–676.2 F1	0.249	0.016
Richness	Y = 10.2–0.2 F1	0.294	0.004

F1 was positively related to the total nitrogen, nitrate nitrogen, total phosphorus and soluble reactive phosphorus.

**Table 3:** Results of forward selection and Monte Carlo permutation tests from the redundancy analysis (RDA) of all 94 sampling sites.

Variables	Lambda-1	*P*	Lambda A	*P*
TN	0.05	0.002^**^	0.05	0.002^**^
Latitude	0.03	0.008^**^	0.02	0.010^*^
T	0.03	0.026^*^	0.03	0.020^*^
NO_3_-N	0.02	0.176	0.02	0.152
Distance	0.01	0.556	0.01	0.370
Crustacea	0.01	0.888	0.00	0.500
NH_4_-N	0.03	0.034^*^	0.01	0.624
Longtitude	0.02	0.120	0.01	0.818
Protozoa	0.00	0.802	0.00	0.706
SD	0.01	0.546	0.01	0.784
TP	0.03	0.010^**^	0.00	0.992
SRP	0.03	0.018^*^	0.01	0.610
Altitude	0.01	0.178	0.00	1.000

Environmental variables were listed by the order of their inclusion in the RDA model. Lambda-1 represents the independent effect of each environmental variable when the variable was treated separately. Lambda A represents the effect that each variable brings in addition to the all variables already selected and the most important variable included first. The *P*-value indicates the significance of each variable either when considered independently or dependently (*P < 0.05, **P < 0.01). TN = total nitrogen; T = water temperature; NO_3_-N = nitrate nitrogen; NH_4_-N = ammonia nitrogen; distance = average geographical distance of a given sampling site from the other sampling sites; Crustacea = total abundance of all crustacean species; Protozoa = total abundance of all protozoa species; SD = Secchi disk depth; TP = total phosphorus; SRP = soluble reactive phosphorus.
