# Supplementary material for: Determinants of community structure of zooplankton in heavily polluted river ecosystems
Source: Sci Rep. 2016 Feb 25;6:22043. doi: 10.1038/srep22043 (PMC4766567; doi:10.1038/srep22043)
Supplement: Supplementary Information [file srep22043-s1.pdf]

Determinants of community structure of zooplankton in heavily polluted river ecosystems

Wei Xiong[1#], Jie Li[2#], Yiyong Chen[1], Baoqing Shan[1], Weimin Wang[2], Aibin Zhan[1*]

[1]Research Center for Eco-Environmental Sciences, Chinese Academy of Sciences, 18 Shuangqing Road, Haidian District, Beijing 100085, China

[2] College of Fisheries, Huazhong Agricultural University, 1 Shizishan Street, Wuhan 430070, China

***.Corresponding author:** Prof. Aibin Zhan; Email: azhan@rcees.ac.cn or zhanaibin@hotmail.com; Phone: (+86)-10-6284-9882; Fax: (+86)-10-6284-9882

[#] These two authors contribute equally to this work

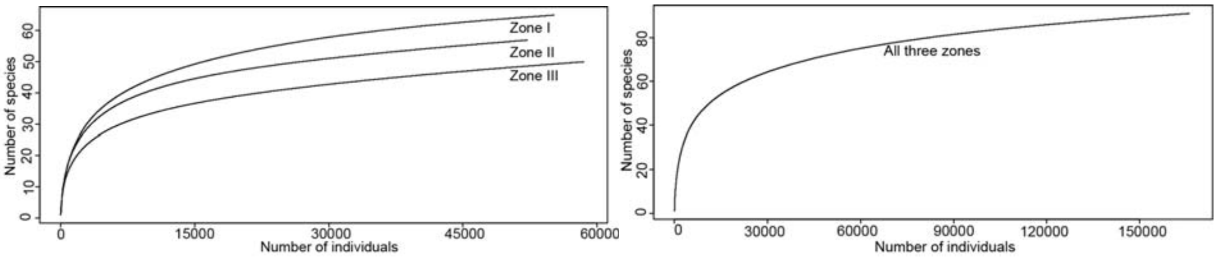

Supplementary Figure S1 Rarefaction curves of counted individuals of rotifers. Left - the each zone, right - the combined set 94 samples.

Supplementary Table S1 Summary of principal component analyses on 13 biotic and abiotic factors in all 94 sampling sites. Factors in red were examined to be representing primary gradients of corresponding component. Distance = average geographical distance of a given sampling site from the other sampling sites, T = water temperature, SD = Secchi disk depth, TP = total phosphorus, SRP = soluble reactive phosphorus, TN = total nitrogen, NO3-N = nitrate nitrogen, NH4-N = ammonia nitrogen, Protozoa = the total abundance of all protozoa species, Crustacea = the total abundance of all crustacean species.

	Component				
	1	2	3	4	5
Longitude	-0.056	0.933	-0.109	-0.068	0.061
Latitude	0.107	0.862	0.296	0.21	0.047
Altitude	-0.233	-0.678	0.516	0.059	-0.097
Distance	-0.388	-0.575	-0.016	0.465	0.222
T	-0.02	-0.16	-0.832	-0.183	-0.11
SD	-0.142	-0.15	0.681	-0.166	-0.101
TP	0.918	0.135	-0.05	0.14	-0.066
SRP	0.912	0.126	-0.059	0.153	-0.089
TN	0.904	0.072	0.009	0.159	0.105
NO_3-N	0.805	-0.126	-0.11	-0.227	0.106
NH_4-N	0.249	0.02	0.025	0.839	-0.055
Protozoa	-0.007	0.072	-0.031	-0.01	0.921
Crustacea	-0.196	0.405	-0.301	0.298	-0.333

Supplementary Table S3 The average similarity of rotifer communities among sampling sites within each zone based on the similarity percentage (SIMPER) analysis in the plain region of the Haihe River Basin.

**Zone I: Average similarity: 13.44**					
Species	Average abundance	Average similarity	Similarity	Contribution (%)	Cumulation (%)
Polyarthra trigla	463.39	2.56	0.45	19.02	19.02
Brachionus angularis	92.68	1.79	0.42	13.31	32.34
Brachionus urceus	330	1.76	0.48	13.11	45.44
Filinia maio	157.5	1.75	0.37	13.04	58.48
Pompholyx sulcata	26.25	0.95	0.32	7.05	65.53
Brachionus capsuliflorus	20.36	0.78	0.32	5.77	71.3
Trichocerca sp.	202.5	0.65	0.32	4.81	76.11
Brachionus calyciflorus	83.57	0.53	0.33	3.97	80.08
Philodina sp.	12.86	0.52	0.24	3.83	83.91
Brachionus budapesllensis	26.25	0.38	0.24	2.85	86.76
Euchlanis sp.	9.11	0.3	0.18	2.21	88.98
Philodina megalotrocha	35.36	0.3	0.12	2.21	91.18
**Zone II: Average similarity: 19.22**					
Brachionus angularis	192.32	4.58	0.78	23.81	23.81
Polyarthra trigla	445.18	3.63	0.58	18.87	42.67
Filinia maio	71.79	2.62	0.49	13.65	56.33
Brachionus calyciflorus	260.89	2.49	0.55	12.93	69.26
Brachionus urceus	73.93	1.93	0.4	10.02	79.28
Keratella valga	26.25	0.71	0.37	3.68	82.96
Filinia longisela	221.79	0.66	0.2	3.45	86.42
Trichocerca pusilla	56.79	0.64	0.28	3.31	89.73
Trichocerca sp.	71.25	0.56	0.26	2.9	92.63
**Zone III: Average similarity: 24.49**					
Brachionus urceus	131.05	5.83	0.81	23.8	23.8
Brachionus angularis	195.79	4.93	0.79	20.13	43.93
Polyarthra trigla	428.29	3.92	0.65	16.02	59.95
Trichocerca sp.	137.76	2.86	0.52	11.67	71.62
Brachionus calyciflorus	123.55	2.32	0.54	9.47	81.09
Filinia maio	112.5	1.29	0.35	5.28	86.37
Brachionus budapesllensis	24.87	0.65	0.29	2.67	89.03
Keratella valga	41.05	0.58	0.3	2.38	91.41
